# Evaluation of Trap Devices for Mass Trapping of *Ceratitis capitata* (Diptera: Tephritidae) Populations

**DOI:** 10.3390/insects13100941

**Published:** 2022-10-17

**Authors:** Marco Colacci, Pasquale Trematerra, Andrea Sciarretta

**Affiliations:** Department of Agricultural, Environmental and Food Sciences, University of Molise, Via de Sanctis, I-86100 Campobasso, Italy

**Keywords:** Mediterranean fruit fly, attractants, pest control, captures

## Abstract

**Simple Summary:**

The Mediterranean fruit fly, *Ceratitis capitata*, is considered one of the world’s most damaging pests, able to infest the fruits of over 300 plant species and adapt to a wide range of climatic zones. It can attack commercially important fruits and losses caused by this pest can be enormous without appropriate control measures. To contribute to the management of Mediterranean fruit fly infestations, in the present paper, we report the effectiveness of commercially available trapping devices (Decis Trap, Tephri Trap Ecological and Conetrap) baited with two types of female attractants (Econex Trypack and Biodelear). The performance of trapping devices was evaluated in semi-field cages in different thermal conditions. Our results showed that the Tephri Trap baited with Trypack or Biodelear and the Decis Trap reported the same performance in capturing females and could all be used for medfly mass trapping. The mass trapping technique can be considered an eco-friendly method to use in combination with other control systems.

**Abstract:**

The Mediterranean fruit fly (medfly), *Ceratitis capitata*, is a key pest of fruit crops in a wide range of climatic zone. Its economic importance is increasing due to its invasion and establishment in new geographical areas caused by global warming. Generally, the management of medfly infestation has been carried out with the use of synthetic pyrethroids and phosphorganic insecticides. Other containment approaches include attract-and-kill, biological control, and the sterile insect technique. The mass trapping technique can be considered an eco-friendly method to use in combination with other control systems. The present study reported the trapping effectiveness of commercially available devices (Decis Trap, Tephri Trap Ecological, and Conetrap) baited with different types of female attractants (Econex Trypack and Biodelear) under different thermal conditions. The performance of trapping systems was evaluated in semi-field cages. Our results showed that the combination of Conetrap with Biodelear was not proven effective, while the Tephri Trap baited with Trypack or Biodelear and the Decis Trap reported the same performance in capturing females and could be used for medfly mass trapping.

## 1. Introduction

The Mediterranean fruit fly (medfly), *Ceratitis capitata* (Wiedemann) (Diptera: Tephritidae), is considered one of the world’s most damaging pests, able to infest the fruits of over 300 plant species and adapt to a wide range of climatic zones [1,2]. Native to sub-Saharan Africa, it is currently a cosmopolitan species present year-round in tropical and subtropical regions of all continents and with a seasonal establishment in temperate zones [3]. Due to climate change, recent scenarios predict a northward expansion of the distribution area in Europe by 42% in 2050 compared to the 2020 presence data [4]. Current knowledge attests to stable populations further north in southern France and northern Italy [3,5,6]. 

*C. capitata* can attack commercially important fruits and losses caused by this pest can be enormous without appropriate control measures. It causes considerable economic damage, estimated at over 2 billion US dollars per year [7]. In the case of stone fruits, medfly can lead to a 100% destruction of production [8].

*C. capitata* is a European and Mediterranean Plant Protection Organization (EPPO) A2 quarantine pest. The economic importance of this insect is significant to the point that in some countries (e.g., mainland USA and Japan) it is subject to very strict phytosanitary regulations that impose barriers and continuous controls on imported fruit to prevent the introduction of potentially risky specimens to lead to infestation outbreaks [3].

Generally, the management of *C. capitata* infestation has been carried out with the use of synthetic pyrethroids and phosphorganic insecticides. Baits, consisting of an attractive substance and a contact insecticide, can be used to contain the level of infestation. Other containment approaches include mass trapping, attract-and-kill, biological control, and the sterile insect technique (SIT) [9,10,11,12].

The first references to mass trapping applications for tephritid fruit flies date back to the early 1920s [13]. As is known, in general, mass trapping consists in placing a large number of traps in the field to reduce the population levels of the target species [14]. These traps can be baited with different types of semiochemicals or food attractants, acting on one or both sexes [15]. Stand-alone mass trapping has been tested to control a wide range of insect pests with different results. In some cases, it showed a significant reduction in target pest population density or pest damage, and in others, it has been ineffective [16]. Several factors, such as attractant trap competitiveness with wild females, trap designs and density, population density, biology and ecology of the target pest, isolation and risk of immigration, influence the success of this technique [15,16].

Against fruit flies, a trapping strategy focused on female attractants is always more efficient as female population reduction can be directly related to fruit damage reduction. For this reason, research on synthetic female attractants and the development of traps focused on females are the key points in the development of the mass trapping system [13]. In the 1990s, studies coordinated by the International Atomic Energy Agency (IAEA) developed a synthetic attractant targeting females composed of three components (ammonium acetate, putrescine and trimethylamine), commercially available as BioLure and usable for medfly monitoring and mass trapping [17,18,19]. Similar products have been developed in recent years and used in medfly management (e.g., Trypack^®^ and Ferag^®^ CC D TM) [20,21,22,23]. Recent studies revealed that adult medflies are attracted to a novel product named Biodelear [24], an attractant derived from a controlled Maillard reaction between fructose and urea in the presence of water [25].

Research has contributed to the development of new trap shapes, colors, attractant combinations, and retention methods to improve trapping performance. Several studies about the medfly-catching performance of different commercial trapping devices can be found in the literature [9,17,26,27]. However, there is limited information about the performance of trapping devices under various temperature conditions [28].

To contribute to the management of Mediterranean fruit fly infestations, in the present paper, we report the effectiveness of commercially available trapping devices baited with two types of female attractants under different thermal conditions. The performance of trapping devices was evaluated in semi-field conditions. The use of field cages represents a good compromise between laboratory and outdoor studies [29].

## 2. Materials and Methods

### 2.1. Insects

A wild-type medfly population, consisting of F_2_ and F_3_ laboratory generations from field-infested fruits collected in Central Italy, was used. Infested apples were collected in November-December 2019 (for the 2020 trials) and November-December 2020 (for the 2021 trials) and transferred into the Laboratory of Applied Entomology at the University of Molise (Campobasso, Italy) in ambient room conditions. The fruits were placed in sterile sand and sifted at regular intervals (twice a week) to collect the pupae. Pupae remained in the same conditions until adult emergence. Upon emergence, adults were placed in plastic-screen cages 40 × 40 × 70 cm with ad libitum access to adult diet (sugar:yeast in ratio 4:1) and water [30]. Flies were allowed to oviposit on banana fruits and the larvae completed their development on them.

One day before release in the field cages, adults 7–20 days old were collected and placed into plastic-screen cages 30 × 30 × 30 cm in groups of 50 individuals (25 males and 25 females) with ad libitum access to sugar and water.

### 2.2. Field Cages

Trapping trials were performed using four cube plastic-screen field cages (2 × 2 × 2 m). In the center of each, a mandarin tree (*Citrus reticulata* Blanco) approximately 1.80 m high and 50 cm in canopy diameter was placed. All the fruits were removed from the trees. Field cages were partially protected from direct sunlight by nearby trees.

### 2.3. Trapping Devices

**Figure 1:**
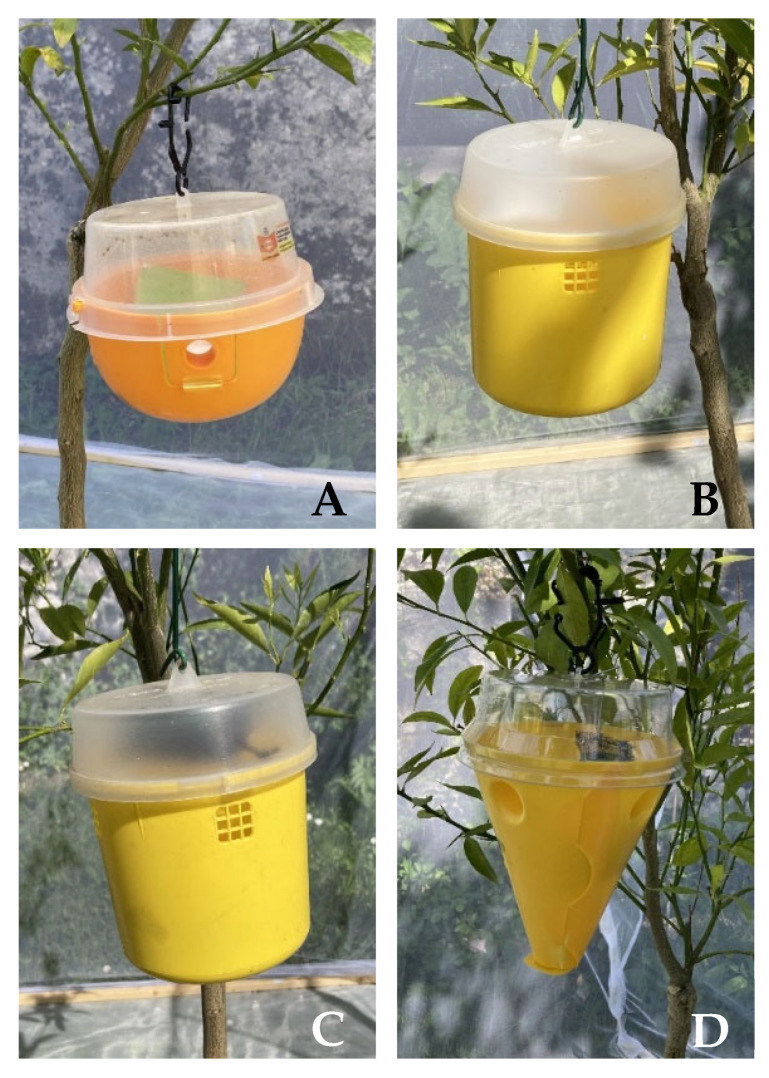
Medfly trapping systems used for the experimentation: Decis Trap (**A**), Tephri Trap Ecological baited with Trypack (**B**), Tephri Trap Ecological baited with Biodelear (**C**), Conetrap baited with Biodelear (**D**).

The following four medfly trapping systems were used for the experimentation:

- Decis^®^ Trap (Bayer CropScience, Milan, Italy) with its attractant (Figure 1A). The Decis^®^ Trap is a ready-to-use trap, which consists of a half-sphere base (14 cm in diameter), on which the dispenser impregnated with food attractants is placed, and a transparent top part treated on the inner surface with insecticide (0.015 g deltamethrin). The base is orange with four lateral holes (2 cm in diameter each) that serve as entrances into the trap. The food attractants are a mixture of 7.8 g ammonium acetate, 0.5 g chlorohydrate trimethylamine and 0.003 g 1,5-diamineopentane [28].
- Tephri^®^ Trap Ecological (Sorygar, Madrid, Spain) baited with a food attractant dispenser Econex Trypack^®^ (Sanidad Agrícola Econex S.L., Murcia, Spain) composed of a mixture of 6.6 g ammonium acetate, 0.7 g trimethylamine hydrochloride and 0.1 g putrescine (1,4-diaminobutane). The trap consists of a cylinder yellow invaginated base (11 cm deep and 12 cm in diameter) with an opaque lid (3.5 cm high). It has five entry holes, one in the invagination of the bottom (3 cm in diameter) and four laterals (2.1 cm in diameter). All holes have a net screen that avoids the entrance of bigger insects (Figure 1B). To capture and retain the attracted insect, 200 mL of water with 2% detergent as a surfactant agent were used.
- Tephri^®^ Trap Ecological baited with a food attractants dispenser Biodelear (Figure 1C). The dispenser consists of piece of Wattex sponge impregnated with 17 g of Biodelear liquid. Moreover, in this case, to capture and retain the attracted insect, 200 mL of water with 2% detergent as a surfactant agent were used.
- Conetrap (Probodelt Estudios Bioagrarios, Amposta, Spain) baited with a Biodelear dispenser (Figure 1D). The traps have a yellow conical base (16 cm high) with four entry holes (2.5 cm diameter) and a transparent lid treated on the inner surface with insecticide (0.0075 g lambda-cyhalothrin). The diameter at the junction of the lid and base is 13 cm. Usually, the trap is provided with its attractant, a mix of hydrolysed proteins. However, in the present trial, we intended to test the trap with a different attractant, Biodelear.

### 2.4. Experimental Design

Semi-field cage trials were conducted in 24 replications from 4 June to 19 July 2020 (12 replications) and from 25 April to 23 May 2021 (12 replications).

For each trial, 50 adults (25 males and 25 females) were released into each field cage with one device. Flies were released at 9:30 am and a trap device with the corresponding attractant was placed on the *C. reticulata* tree at a height of approximately 1.20 m at 10:00 am. Flies had ad libitum access to food source (granulated sugar) and water (water-soaked cotton pads placed on the branches of the trees).

Medflies trapped (number of flies per sex) were recorded at hourly intervals from 11:00 am to 6:00 pm (8 records). Twenty-four replicates were run (trial days) under different temperature conditions. Trapping devices were rotated within field cages in a clockwise manner per replication to minimize the influence of the cage location. 

The temperature and relative humidity were recorded by a data logger placed in a field cage.

At the end of each trial day, the cages were cleaned of insects and wiped. The free flies in the cages were counted to make sure no flies were left in the cage.

### 2.5. Data Analysis

The Kolmogorov–Smirnov test was used to assess the normality of data distributions. Generalized linear models (GLMs) with Poisson loglinear distribution were used to evaluate the effects of treatment (mass trapping device), sex, temperature, and their interactions (excluding treatment × sex × temperature) on adult captures. 

One-way ANOVA followed by the post-hoc Tukey HSD test was used to determine the effect of treatment on total, males and females captures and on the proportion of females captured, estimated by dividing the total female captures by the total individuals (males + females). Before the analyses were performed, data were log (x + 1) transformed to normalize variance and standardize means. 

Statistical analyses were performed using SPSS (v.26.0) statistical software (SPSS Inc., Chicago, IL, USA).

## 3. Results

### 3.1. Climatic Data

**Figure 2:**
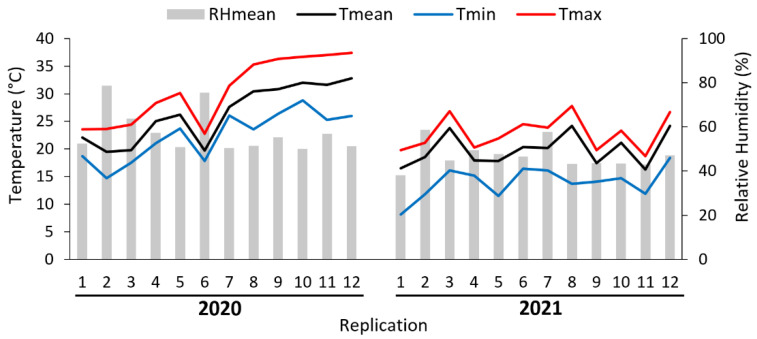
Mean (Tmean), minimum (Tmin), maximum (Tmax) temperatures and mean relative humidity (RHmean) during semi-field cage trials.

The average temperature inside the field cages ranged from 16.5 to 32.8 °C. The absolute minimum and maximum temperature ranged from 8.1 to 28.8 °C and 18.7 to 37.4 °C, respectively. The mean relative humidity ranged from 48% to 78% (Figure 2). 

### 3.2. Efficacy of Trapping Devices

The GLM revealed that some parameters were significant predictors of medfly adult captures (Table 1).

The GLM showed that trapping devices had different efficacies in medfly adult capture (χ^2^ = 83.865, df = 3, *p* < 0.001), sex ratio capture (χ^2^ = 9.341, df = 1, *p* = 0.002), and temperature (χ^2^ = 64.616, df = 22, *p* < 0.001).

The interaction of trapping device with sex (χ^2^ = 8.749, df = 3, *p* = 0.033) and temperature (χ^2^ = 106.319, df = 62, *p* < 0.001) were both significant, this suggest different sex ratio of the captured adults among trapping devices and that their performance was affected by temperature. 

The interaction between sex and temperature was not significant (χ^2^ = 22.125, df = 22, *p* = 0.452), suggesting a similar sex ratio of the captured adults at different temperatures. 

Tephri Traps baited with Trypack captured the highest number of adults followed by Tephri Traps baited with Biodelear, Decis Traps and Conetraps baited with Biodelear (Table 2). Statistically, the least effective trap was Conetrap baited with Biodelear (ANOVA, F = 26.918, df = 3, *p* < 0.001) while the other three traps showed no statistical difference in catching capacity.

The trapping device was a significant predictor of the male (ANOVA, F = 19.719, df = 3, *p* < 0.001) and female (ANOVA, F = 25.924, df = 3, *p* < 0.001) captures. 

The male numbers captured in Thepri trap with Biodelear were significantly higher than that in Decis Trap and Conetrap with Biodelear. There was no significant difference in male captures between the Decis Trap and Thepri Trap with Trypack and between the latter and the Thepri trap with Biodelear (Table 2).

For female captures, the most effective device was the Tephri Trap baited with Trypack; no statistical differences between the Tephri Trap with Biodelear and Decis Trap were observed. Conetrap with Biodelear captured statistically fewer females than the other three trap devices (Table 2).

During the trials, in proportion, more females than males were captured in all trapping devices except for the Tephri Trap baited with Biodelear, with no statistical differences among them (ANOVA, F = 1.967, df = 3, *p* = 0.125) (Table 2).

**Figure 3:**
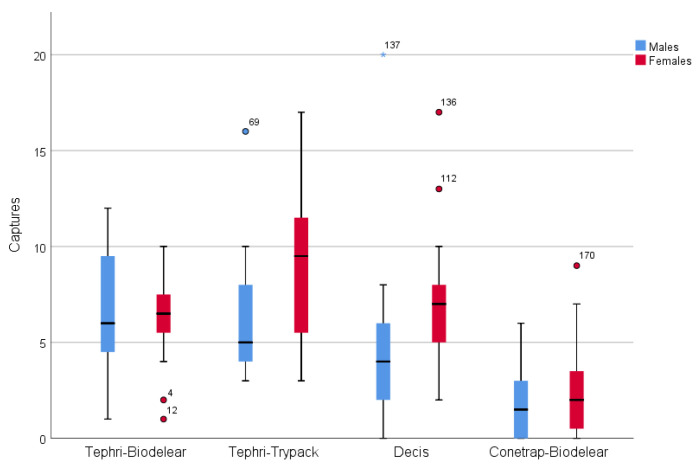
Box plot (horizontal line = medians; box = quartiles; vertical line = Q1 – 1.5 × (inter quartile range) and Q3 + 1.5 × (inter quartile range); dots = outliers; asterisk = extreme outliers) depicting the number of males and females caught in the four trapping devices in the semi-field cage trials.

Figure 3 gives the male and female captures for each trapping device during field cage trials. The graph depicts the higher performance of the Tephri Traps baited with Trypack and with Biodelear and of the Decis Trap and the lower performance of the Conetrap baited with Biodelear.

**Figure 4:**
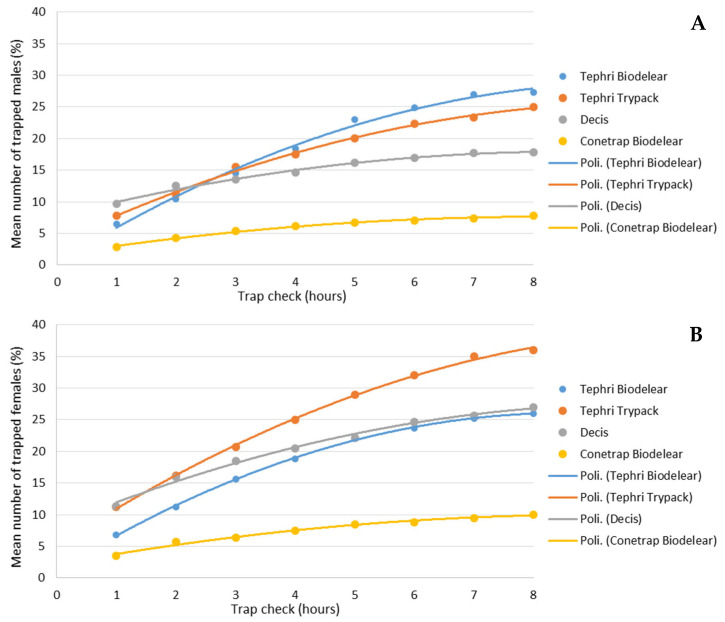
Cumulative captures of male (**A**) and female (**B**) catches (in %) over the 8-h trial period in the four trapping devices.

Figure 4 shows the cumulative effect of the different traps over the 8 h of trials. The average percentage of captured male adults was between 7.8% in Conetrap baited with Biolure and 27.3% in Thepri traps baited with Biodelear (Figure 4A). Higher catch rates were recorded in the capture of adult females. In this case, after 8 h, recapture rates ranged from 10% in Conetrap baited with Biolure to 36% in Thepri traps baited with Trypack (Figure 4B).

**Figure 5:**
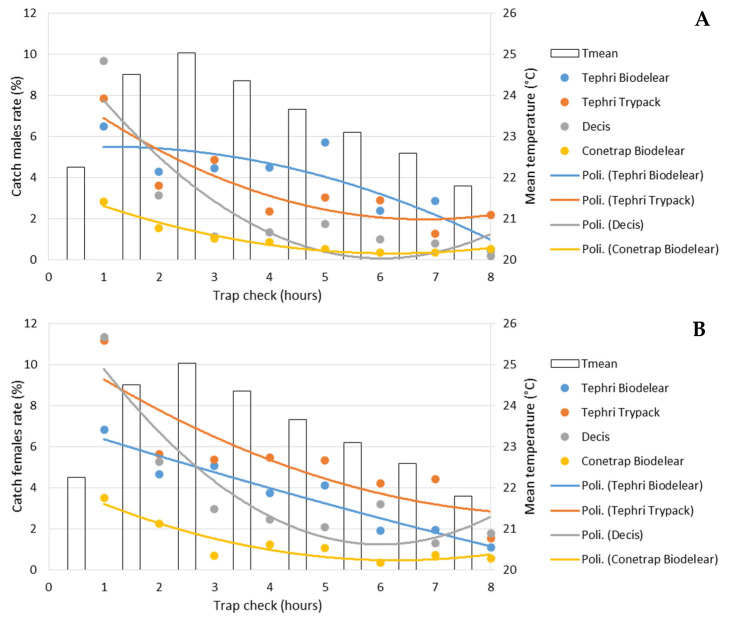
Performance of male (**A**) and female (**B**) catches (as %) in the 8 checks in the four trapping devices and mean temperature recorded.

Figure 5 shows the percentage of male (Figure 5A) and female (Figure 5B) catches, detected in the different traps during each control. All traps recorded their highest catch rates in the first check (after one hour of testing), when Decis Trap recorded the highest capture rate for both males (9.7%) and females (11.3%) compared to the other traps. 

The catching efficiency of the traps decreased throughout the trap checks for Tephri Traps baited with Biodelear and Trypack, and Conetrap baited with Biodelear, while the Decis Trap, mainly for females, registered an increase in catches during the last controls.

## 4. Discussion

Mass trapping has been shown to be an effective pest management tool against medflies. The use of female attractants in trap devices allowed this technique to be applied effectively in reducing the adult population and fruit damage [13]. Several studies have reported a good efficacy of this control tool in different fruit orchards and countries [31,32,33,34]. Instead, low effectiveness of the mass trapping technique was found in orchards with high medfly population density [21,35]. Another limitation in the practical use of mass trapping may be the cost [13]. For example, in the control of medfly, the 2020 cost of mass trapping in Italy orchards (assessed for three months of application with a density of 100 traps/ha, including labor in preparation and trap deployment and one time servicing) ranges from 557 euro to 737 euro using Decis Trap or Tephri Trap baited with Trypack, respectively. These costs are more expensive than pest management carried out using deltamethrin (38 euro/ha per treatment) or fosmet (84 euro/ha per treatment) [36]. 

Because the mass trapping cost is highly dependent on the number of traps required, the selection of effective and easy-to-manage trap–attractant combinations, which might allow a reduction of the trap density and management time required to achieve fruit protection, is essential for the successful adoption of this technique. According to results obtained by our trials, Tephri Traps baited with Biodelear or Trypack and Decis Trap reported the same trapping effectiveness on adult medfly females. Regarding the capture of males, Tephri Traps with both attractants performed better than the other two tested traps.

The Conetrap baited with Biodelear was found to be the least effective in catching among the tested traps. Broughton and Rahman [27] reported a good efficacy of Conetrap baited with BioLure. Similar results were confirmed also by Tlemsani and Boulahia-Khedr [37] with a field trial in citrus orchards baiting the Conetrap with dry food attractants (ammonium acetate, trimethyl amine and alkaline diamine formulated in a single dispenser). In our trial, the combination Conetrap–Biodelear did not produce good results. However, when installed in the Tephri Trap, the Biodelear showed good trapping efficiency. In this case, according to Miranda et al. [38], the humid environment within the Tephri Trap may have facilitated the emission of the attractant.

The two attractants, Trypack and Biodelear, when placed in the same trap type (Tephri Trap) in semi-field cage trials showed the same trapping efficiency against male and female medflies. Moreover, Kouloussis et al. [24], in field trials using McPhail-type traps, reported that Biodelear performed satisfactorily and in most cases proved equally effective to the BioLure in medfly capture. 

Following our results, Bali et al. [28] reported the same trapping efficacy between the Tephri Trap baited with BioLure and the same trap baited with Biodelear in spring trials performed in a field cage. However, the same authors reported lower effectiveness of the Decis Trap in spring field cage trials compared to the Tephri Traps baited with BioLure or Biodelear and to the International Pheromone McPhail traps (IPMT) baited with BioLure or Biodelear. As opposed to this, in our trial, the Decis Trap performed the same as the Tephri Trap baited with both attractants. 

The capacity of the trap to catch females is one of the requirements for successful mass trapping. In our trial, more females were captured in all trapping devices except for the Tephri Trap baited with Biodelear, which reported a proportion of 1:1. In addition, our results revealed that the female selectivity of the four traps is not affected by temperature. These data differ from field cages experiment and field trials, conducted respectively in Greece and Spain, reporting that at low temperatures more males were caught than females [28,38].

The highest average proportion of captured adults during the trial was 27.3% of the released males (recorded in Tephri Traps baited with Biodelear) and 36% of released females (recorded in Tephri Traps baited with Trypack). The proportion of captures never exceeds 56% of the released individuals, as recorded in the Decis Trap. As also reported by Bali et al. [28], this indicates that a considerable proportion of individuals of both sexes were not trapped by the devices also under confinement in cages. Low recapture rates explain the better performance of the mass trapping technique in cases where medfly population density is low [35].

For both sexes, the catches trend showed that the highest percentages of captures occurred in the first check, after one hour of medfly-trap interaction, suggesting that, many of the caught individuals respond immediately. In the first check, the Decis Trap recorded the highest catch percentages for both sexes compared to the other three traps. The catching efficiency of the traps decreases throughout the time for Tephri Traps baited with Biodelear or Trypack and Conetrap baited with Biodelear, whereas the Decis Trap registered an increase in female catches during the last checks when the average temperature dropped. 

Considering the overall efficacy of trapping devices considered in our experimentation, the Tephri Trap baited with Trypack or Biodelear and Decis Trap could be used for thje mass trapping of medfly. However, traps with a liquid retention system (for example Tephri Trap), when used in a large number in the field, necessitate considerable management efforts (e.g., transport of the liquid and evaporated liquid refilling), resulting in increasing costs of application and handling. 

## 5. Conclusions

Our study in semi-field conditions showed that the Tephri Trap baited with Trypack or Biodelear and Decis Trap reported the same efficacy in capturing females and could all be used for medfly mass trapping. On the contrary, the combination of Conetrap with Biodelear was not proven effective, so this trap should be used with its attractant, a mix of hydrolysed proteins. However, because the field cage trials represent a compromise between laboratory and field experiments [29], the results obtained in semi-field cages should ideally be confirmed by field tests.

## Figures and Tables

**Table 1:** Results of the GLMs on the performance of the trapping device, sex, temperature and two-way interactions on the captures in the semi-field cage trials.

Parameter	χ^2^	df	*p*-Value
Trapping device	83.865	3	<0.001
Sex	9.341	1	0.002
Temperature	64.616	22	<0.001
Trapping device × Sex	8.749	3	0.033
Trapping device × Temperature	106.319	62	<0.001
Sex × Temperature	22.125	22	0.452

**Table 2:** Captures of medfly adults (±SE) and the proportion of females (±SE) in the four trapping devices.

Trapping Devices	Average Number of Captures (Males + Females)	Average Number of Male Captures	Average Number of Females Captures	Average Proportion of Females (Females/(Males + Females)
Tephri trap-Biodelear	13.33 ± 0.96 ^a^	6.83 ± 0.61 ^a^	6.50 ± 0.45 ^a^	0.49 ± 0.02 ^a^
Tephri trap-Trypack	15.25 ± 1.21 ^a^	6.25 ± 0.61 ^ab^	9.00 ± 0.83 ^a^	0.57 ± 0.03 ^a^
Decis trap	11.33 ± 1.24 ^a^	4.58 ± 0.80 ^b^	6.75 ± 0.67 ^a^	0.63 ± 0.04 ^a^
Conetrap-Biodelear	4.42 ± 0.80 ^b^	2.00 ± 0.39 ^c^	2.42 ± 0.49 ^b^	0.57 ± 0.07 ^a^

In the same column, means followed by the same letter are not significantly different at 5% level in each season (Tukey HSD test, *p* < 0.05).

## Data Availability

The datasets used or analyzed during the current study, are available upon request, from the FF-IPM project.
